# CLPP-Null Eukaryotes with Excess Heme Biosynthesis Show Reduced L-arginine Levels, Probably via CLPX-Mediated OAT Activation

**DOI:** 10.3390/biom14020241

**Published:** 2024-02-19

**Authors:** Jana Key, Suzana Gispert, Arvind Reddy Kandi, Daniela Heinz, Andrea Hamann, Heinz D. Osiewacz, David Meierhofer, Georg Auburger

**Affiliations:** 1 Goethe University Frankfurt, University Hospital, Clinic of Neurology, Experimental Neurology, Heinrich Hoffmann Str. 7, 60590 Frankfurt am Main, Germany; jana.key@kgu.de (J.K.); gispert-sanchez@em.uni-frankfurt.de (S.G.); s8136159@stud.uni-frankfurt.de (A.R.K.); 2 Institute of Molecular Biosciences, Faculty of Biosciences, Goethe-University Frankfurt, 60438 Frankfurt am Main, Germany; dheinz089@gmail.com (D.H.); a.hamann@bio.uni-frankfurt.de (A.H.); osiewacz@bio.uni-frankfurt.de (H.D.O.); 3 Max Planck Institute for Molecular Genetics, Ihnestraße 63-73, 14195 Berlin, Germany; meierhof@molgen.mpg.de

**Keywords:** Perrault syndrome, PLP, metal homeostasis, delta amino acids

## Abstract

The serine peptidase CLPP is conserved among bacteria, chloroplasts, and mitochondria. In humans and mice, its loss causes Perrault syndrome, which presents with growth deficits, infertility, deafness, and ataxia. In the filamentous fungus *Podospora anserina*, CLPP loss leads to longevity. CLPP substrates are selected by CLPX, an AAA+ unfoldase. CLPX is known to target delta-aminolevulinic acid synthase (ALAS) to promote pyridoxal phosphate (PLP) binding. CLPX may also influence cofactor association with other enzymes. Here, the evaluation of *P. anserina* metabolomics highlighted a reduction in arginine/histidine levels. In *Mus musculus* cerebellum, reductions in arginine/histidine and citrulline occurred with a concomitant accumulation of the heme precursor protoporphyrin IX. This suggests that the increased biosynthesis of 5-carbon (C5) chain deltaALA consumes not only C4 succinyl-CoA and C1 glycine but also specific C5 delta amino acids. As enzymes responsible for these effects, the elevated abundance of CLPX and ALAS is paralleled by increased OAT (PLP-dependent, ornithine delta-aminotransferase) levels. Possibly as a consequence of altered C1 metabolism, the proteome profiles of *P. anserina* CLPP-null cells showed strong accumulation of a methyltransferase and two mitoribosomal large subunit factors. The reduced histidine levels may explain the previously observed metal interaction problems. As the main nitrogen-storing metabolite, a deficiency in arginine would affect the urea cycle and polyamine synthesis. Supplementation of arginine and histidine might rescue the growth deficits of CLPP-mutant patients.

## 1. Introduction

Bacterial ClpP (ATP-dependent caseinolytic serine peptidase, proteolytic subunit, also named “endopeptidase Ti” in *Escherichia coli* bacteria) cleaves peptide bonds between carboxy groups and amino groups. When ATP hydrolysis by an associated AAA+ motor domain translocates several residues of substrate proteins into the proteolytic chamber composed of symmetric heptameric ClpP rings, artificial substrates such as the milk emulsifier protein casein can be cleaved in the presence of magnesium [1,2,3,4,5]. The strong conservation of ClpP (around 50% amino acid identity [6]) from bacteria to chloroplasts and mitochondria has enabled the unequivocal demonstration of its crucial role in cell stress responses [7,8]. However, its degradation targets are still under debate given that bulk protein breakdown in mitochondria depends more on LonP than ClpP orthologs across evolution [9,10]. Further insights into the role of ClpP may be derived from the fact that it depends on an AAA+ disaggregase for substrate selection and ATP hydrolysis. This unfolding and chaperone function is usually performed by ClpX, but in some bacterial strains by ClpA–E. The abundance of this AAA+ disaggregase correlates inversely with ClpP protein levels [7,11,12], so the absence of ClpP function might be compensated by high ClpX activity. 

**Figure 1:**
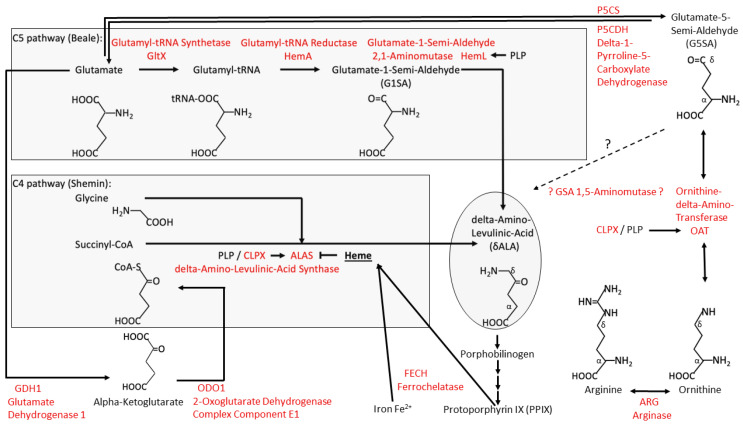
For the mitochondrial biosynthesis of heme from protoporphyrin IX and iron, a symmetric tetrapyrrole ring system has to be generated from the building block delta-aminolevulinic acid (δALA, or simply ALA). The production of this non-cognate amino acid occurs predominantly via the C5 pathway in archaea/plants versus the C4 pathway in fungi/animals. According to the present study, the overactivity of CLPX/ALAS and CLPX/OAT in two models of PRLTS3 pathogenesis reduces arginine, similar to glycine in CLPP-null *P. anserina*. Arginine is more strongly affected than serine in CLPP-null mouse cerebellum, presumably via G5SA consumption. Whether the sequence homology and structural similarity between OAT and GSA aminomutase reflects an ancient processing of ornithine as a C5 substrate for δALA biosynthesis remains to be tested further and is highlighted by question marks. Arrows reflect metabolic flux, and CLPX/PLP activation of ALAS/OAT versus ALAS repression by the end product heme. Enzymes and their protein symbols are shown in red letters. P5CS represents delta-1-pyrroline-5-carboxylate synthase, also known as ALDH18A1 (aldehyde dehydrogenase 18 family member A1).

In *Saccharomyces cerevisiae* yeast, a CLPP ortholog has not been identified, while the ClpX orthologous protein MCX1 is abundant [13]. MCX1 does not contain the conserved IGF domain for CLPP interaction, so yeast CLPX is thought to act purely as an unfoldase/chaperone without associated substrate cleavage [14]. Its function was found to be crucial, mediating the activation of the initial step in heme biosynthesis: MCX1 unfolds the delta-aminolevulinic acid (δALA, deltaALA, or simply ALA) synthase enzyme (ALAS) and catalyzes the incorporation of its cofactor, pyridoxal 5′-phosphate (PLP or P5P, the active form of vitamin B6), which is needed for efficient transamination [14]. δALA constitutes the building block for the tetrapyrrol rings in downstream porphyrins, which can chelate Fe^2+^ or Mg^2+^ to yield heme or chlorophyll, respectively (Figure 1). In archaea/bacteria, δALA is also the building block for the biosynthesis of the corrin rings from their precursor porphobilinogen. Corrin rings can chelate Co^2+^ to yield cobalamin (vitamin B12), a crucial cofactor of enzymes. In mammals, two enzymes require cobalamin binding: methionine synthase and methylmalonyl-CoA mutase. They control mitochondrial C1 metabolism, S-adenosyl-methionine (SAM)-dependent methylations, and epigenetics [15]. 

The role of CLPX in ALAS activation, via the control of PLP association, is highly conserved from yeast to humans. The deletion of yeast CLPX leads to a 5-fold reduction in δALA and to a reduction in heme by half [14]. In the zebrafish *Danio rerio*, phenotypes due to the depletion of CLPX can be rescued by the administration of δALA [16]. In *Homo sapiens*, mutations in CLPX trigger hematological disorders due to errors in heme biosynthesis [17,18]. ALAS1 degradation within human mitochondria was found to depend on heme binding and the CLPXP complex [19]. ALAS synthesizes the non-proteinogenic δALA from succinyl-coenzyme A (succinyl-CoA) and the proteinogenic amino acid glycine (see Figure 1). This biosynthesis pathway involving the five-carbon (C5) compound δALA from the four-carbon (C4) source succinyl-CoA plus glycine (as described by Shemin and coworkers [20]) is predominant in animals, fungi, protozoa, and alphaproteobacteria (ancestors of mitochondria). 

A more ancient mechanism for δALA biosynthesis (defined by Beale and coworkers [21], see Figure 1) uses C5 acids (alpha-ketoglutarate or rather its transaminated form, glutamate). This pathway is prominent in archaea, most bacteria, algae, and plants. Several microorganisms use both C4 and C5 substrates [22,23]. In this C5 pathway, δALA is produced by a chain of three enzymes (GltX, HemA, and HemL), with the final step converting glutamate-1-semi-aldehyde (GSA, or better G1SA) being performed by the PLP-activated enzyme HemL (also known as GSA 2,1-aminomutase, or GSA aminotransferase). There are no HemA orthologs in animals, so δALA production from glutamyl-tRNA cannot occur; therefore, it has been argued that the C5/Beale pathway is not used by eukaryotes. Indeed, the nitrogen from glycine is used for heme biosynthesis in mammals under normal conditions rather than glutamate/GSA according to isotope-labeled metabolism studies [20], but this does not exclude that a pathological overactivation of δALA production due to an excess of CLPX might recruit additional nitrogen sources such as GSA. It is conceivable that HemA became unnecessary in eukaryotes because GSA can be produced from other sources and can be converted to δALA by a homolog of HemL. Bacterial HemL has significant sequence homology in *P. anserina* (PODANS) with PODANS_5_6860 (GSA 2,1-aminomutase), but also PODANS_7_10880 (ornithine delta-aminotransferase or OAT), the aminotransferases PODANS_6_1290, PODANS_7_5560, and PODANS_3_9430, according to the STRING Heidelberg webserver [24]. In mice, the closest HemL homolog is OAT, but it also has significant homology with PHYKPL (5-phosphohydroxy-L-lysine phospholyase), ETNPPL (ethanolamine-phosphate phospholyase), AGXT2 (alanine-glyoxylate aminotransferase 2), and ABAT (4-aminobutyrate aminotransferase), all of which require PLP as a cofactor. We might, therefore, hypothesize that in mammals, OAT-PLP facilitates the production of δALA via GSA that is derived directly from ornithine/arginine rather than more indirectly from glutamate/alpha-ketoglutarate (see Figure 1). Recently, members of our Frankfurt team reported that endogenous OAT and CLPX interact based on coimmunoprecipitation analyses of CLPP-mutant cells and observed a pathological accumulation of iron and cobalt in these cells [25]. Therefore, OAT might be unfolded by CLPX to use PLP in an analogous manner to ALAS, contributing to the excessive production of heme (in eukaryotes) and cobalamin (in archaea/bacteria).

What is PLP needed for? Coexisting with RNA since the prebiotic world [26], PLP became useful in the optimized biosynthesis and degradation of amino acids via its protein association, first as a chaperone [27] and then as a cofactor of enzymes. It is required for all transaminations, some decarboxylations, dehydrations, and racemizations, but it can also modulate desulfurizations [28]. The main role of PLP in the metabolism of cognate proteinogenic amino acids with their amino group at the alpha-carbon position does not appear to depend on CLPX unfolding functions. However, PLP also acts as a key cofactor in the metabolism of non-cognate amino acids that are derived from arginine, e.g., its blood pressure signaling derivatives like alpha-keto-delta-(*N*,*N*-dimethylguanidino)-valeric acid, its non-proteinogenic precursors citrulline/ornithine, and its non-proteinogenic analogue homoarginine. Furthermore, PLP is a cofactor in the generation of non-canonical amino acids that act as (i) detoxifiers (like cystathionine, glutathione, and the polyamines putrescine, spermidine, and spermine, with their derivative hypusine that is required for ribosomal fidelity during translation elongation across poly-proline sequences [29,30]); (ii) messengers (like gamma-amino-butyric acid or GABA); and (iii) toxins (like ibotenic acid) [31,32]. All such metabolites are derived from classical amino acids but have an amino group that is distant from the carboxylic acid group at the delta or gamma position. Therefore, the aminotransferase domain with bound PLP would need to act at a δ- or γ-carbon (see Figure 1 for Arg, Orn amino groups, and for the change of the amino group from the α-carbon in G1SA to the δ-carbon in δALA which would require an aminomutase function). Any enzyme that acts as a transaminase or aminomutase at δ-carbons may need CLPX-mediated unfolding, if it is docked at the carboxy group, to reach across this distance [33]. 

In *Caenorhabditis elegans* nematode worms, CLPP depletion was observed to have a prominent impact on the cellular response to unfolded protein stress within mitochondria (UPR^mt^) [34], a retrograde signaling pathway from mitochondria to the nucleus that regulates transcription factors like ATF4 [35]. Similarly, the overexpression of CLPX in mammalian cells was also reported to activate the UPR^mt^ pathway [36]. As an underlying mechanism in bacteria and mammalian cells, it was shown that the stalling of ribosomal translation triggers a polypeptide chain elongation with an alanine–threonine-rich tail, which is recognized by CLPX and results in the elimination of this misfolded protein fragment by CLPP [37,38,39]. Ribosomal stalling and nascent peptide misfolding are frequent events during protein synthesis, usually due to the presence of poly-proline motifs [40] or other ribosomal translation fidelity problems [41,42]. Thus, the CLPXP pathway has a quality surveillance function for mitochondrial polypeptide chains.

Human data showed that a loss-of-function of CLPP (and rarely a possible gain-of-specific-function within CLPP, see [43]) causes phenotypes classified as Perrault syndrome type 3 (PRLTS3) [44]. The original definition of Perrault syndrome was based on its autosomal recessive inheritance, and on the co-occurrence of primary ovarian insufficiency with subsequent deafness [45]. In addition, PRLTS3 patients show a strong reduction in height [44] and weight, as well as an age-associated widespread nervous system atrophy that results in ataxia, neuropathy, and leukodystrophy [46]. The proteome profiling of PRLTS3 patient fibroblasts confirmed a several-fold CLPX accumulation as the most consistent consequence of the loss of CLPP function [47]. Most of the other causes of Perrault syndrome are due to mutant genes that encode components of the mitochondrial RNA processing or translation pathway, such as the nucleoid replication/repair factor TWNK, the RNA degradation factor PRORP, the tRNA–amino acid synthases HARS2 and LARS2, and the mitoribosomal factor RMND1 [48,49]. It remains unknown how these phenotypes and the mitoribosomal pathology can be mechanistically explained given the specific heme production problems and possibly multiple other CLPX-PLP-associated enzyme alterations.

Small drugs that are in clinical use to counteract bacterial infections were found to inhibit or activate CLPP and were later reported to also affect the viability and proliferative activity of carcinomas [50]. Several CLPP agonists (ONC201, as well as other imipridones) were demonstrated to be quite efficient against not only bulk tumor cells but also cancer stem cells, cancer-associated fibroblasts, and immune cells within the tumor microenvironment. ONC201, as a single agent, is useful for subtypes of high-grade glioma, endometrial cancer, prostate cancer, mantle cell lymphoma, and adrenal tumors [51,52]. Other CLPP agonists are being evaluated for leukemia treatment [53]. Thus, a detailed understanding of the impact of CLPP on mitochondrial metabolism is urgently needed.

In CLPP-null mice used as a PRLTS3 model, both females and males are completely infertile [54], with a growth deficit despite increased food consumption, reduction of body length by 10% and weight by 30%, as well as a reduction of fat mass by 64% and lean mass by 24% [55,56]. Deafness occurs by the age of 1 year [12]. Similar to bacteria and human cells, in mouse tissues, the primary consequence of an absence of CLPP is a >3-fold accumulation of CLPX [12]. The abundance of CLPX increases together with its interacting mitochondrial nucleoid proteins and mtDNA [47], and with ribonucleoproteins, particularly from the mitoribosomal large and small subunit (LSU and SSU) or translation elongation machinery [57]. The consequent mitochondrial dysfunction activates the innate immune defenses and leads to protection from bacterial infections, such as *dermatitis ulcerosa* [12,58,59,60]. Given that the CLPP-null mouse mutant is an authentic model of PRLTS3 patient phenotypes, we used it for in-depth studies of nervous system mitochondrial amino acid metabolism, taking advantage of the fact that the blood–brain barrier shelters the nervous system from the feeding-related fluctuations in nutrient availability. Adult cerebellar tissue was chosen since it is the area where ataxia phenotypes are usually triggered, and because of its uniform structure. To elucidate the consistency of CLPP effects across evolution, this approach was complemented by an analysis of a fungal eukaryote during the early stage (at day 8) of its life cycle. 

The filamentous fungus *Podospora anserina* has been characterized in much detail as a model organism for aging research. Its usefulness is due to its short lifespan (normally around 3–5 weeks), a phenotypically obvious senescence stage, and several isolates with altered survival due to mutations, which can be easily generated by molecular genetic techniques in this organism [61]. In contrast to humans, which are diplonts with two homologous chromosomes and, thus, two alleles for every gene, *P. anserina* is a haplont with a single set of chromosomes, possibly explaining the different phenotypes caused by identical mutations in these two organisms. 

The genetic deletion of CLPP was shown to trigger a 2–3-fold prolongation of healthy lifespan in *P. anserina* (*Pa*), and this effect could be greatly extended by the additional deletion of the i-AAA mitochondrial inner membrane protease (*ΔPaIap*). Human CLPP was able to substitute for PaCLPP and revert the phenotype [62]. The combined deletion of CLPP and AMP kinase (*ΔPaClpP/ΔPaSnf1*) extended the healthy lifespan by more than 500% at 27 °C under constant light for a specific mating type, an effect that was lost upon induction of high-temperature stress [63]. 

Two published reports on *P. anserina* CLPP-null strains underlie the present project. Regarding the metabolomics profile of the *ΔPaClpP* strain after 5 and 20 days of growth, low levels were reported for most amino acids, phosphoenolpyruvate, and ATP/GTP/CTP/UTP at a young age, followed by unexpectedly high levels of these metabolites in later life [63]. In a further study to identify CLPP cleavage substrates, catalytically inactive HsCLPP-S153A was introduced into the *ΔPaClpP* strain; a failure to rescue the phenotype was demonstrated, and 47 mitochondrial proteins were co-purified with wild-type and mutant CLPP as potential interactors, while 19 mitochondrial proteins selectively co-purified with mutant CLPP as putative degradation targets [64]. A bioinformatic gene ontology term analysis (GO term analysis) assigned these potential CLPP interaction partners and substrates to different mitochondrial processes and pathways such as the respiratory chain, TCA cycle, chaperone system, or amino acid metabolism. Strikingly, components of the N-module of respiratory chain complex I appear to be conserved CLPP substrates among species such as *P. anserina*, *Arabidopsis thaliana*, and mice [6,64,65,66].

To elucidate the impact of these findings in more detail and to identify evolutionarily conserved molecular pathways controlled by CLPP, we re-analyzed the *P. anserina* metabolome and protein interaction data and complemented them with global proteome profile data of an 8-day-old CLPP-null *P. anserina* mutant and with data from adult mouse cerebella. Finally, we asked the question of whether the absence of CLPP via excess CLPX might modulate the PLP binding of additional mitochondrial enzymes beyond ALAS, thus modulating the levels of cognate and non-cognate amino acids and, in parallel, TCA cycle intermediate metabolites.

## 2. Materials and Methods

### 2.1. Culture Conditions of P. anserina

The media used in this study are described in [63].

### 2.2. Metabolic Profiling of P. anserina

The metabolic profiling method is described in [63]. In short, four biological replicates of 5- and 20-day-old wild-type and CLPP-null strains were studied by Metabolomic Discoveries (Metabolomic Discoveries GmbH, Potsdam, Germany).

### 2.3. Global Proteome Profile of P. anserina

Wild-type and CLPP-null strains were germinated for two days, grown on M2 medium (for three days), and inoculated in CM liquid medium (0.1% KH_2_PO_4_, 0.05% KCl, 0.05% MgSO_4_, 1% glucose, 0.37% NH_4_Cl, 0.2% tryptone, 0.2% yeast extract, 0.1% stock solution A) for an additional three days with shaking in constant light. Stock solution A consists of 0.1% ZnSO_4_, 0.1% FeCl_2_, 0.1% MnCl_2_, pH 6.5 (KOH). After total protein extraction [63], 20 µg of protein (in 16.5 µL) was mixed with 3.5 µL SDS loading buffer (6% Tris/HCl pH 6.8, 48% glycerol, 9% ß-mercaptoethanol, 0.03% bromophenol blue, 6% SDS). After boiling (10 min, 95 °C) and cooling on ice, the samples were stored at −20 °C. Later, 20 µg of protein were separated in 4–12% gradient Novex Bis-Tris gels (LifeTechnologies, Carlsbad, CA, USA). SDS-PAGE was stopped after the proteins entered the first 0.5 cm of the separating gel. The gels were fixed in 50% (*v*/*v*) methanol, 10% (*v*/*v*) acetic acid, and 10 mM ammonium acetate for 30 min and stained with Coomassie (0.025% Serva Blue G, 10% (*v*/*v*) acetic acid). The gel was cut into 4 sections and collected in 96-filter well plates (30–40 µm PP/PE, Pall Corporation, Port Washington, NY, USA). The gel pieces were destained in 60% methanol and 50 mM ammonium bicarbonate (ABC). The solutions were removed by means of centrifugation for 2 min at 600 g. The proteins were reduced in 10 mM DTT and 50 mM ABC for one hour at 56 °C, and alkylated for 45 min in 30 mM iodoacetamide. The samples were digested for 16 h with trypsin (sequencing grade, Promega, Madison, WI, USA) at 37 °C in 50 mM ABC, 0.01% Protease Max (Promega), and 1 mM CaCl_2_. The peptides were eluted in 30% acetonitrile and 3% formic acid, centrifuged into a fresh 96-well plate, dried in a speed vac, and resolved in 1% acetonitrile and 0.5% formic acid. 

Liquid chromatography/mass spectrometry (LC/MS) was performed on a Thermo Scientific™ Q Exactive Plus equipped with an ultra-high performance liquid chromatography unit (Thermo Scientific Dionex Ultimate 3000) and a Nanospray Flex Ion Source (Thermo Scientific, Waltham, MA, USA). The peptides were loaded on a C18 reversed-phase precolumn (Thermo Scientific) followed by separation with 2.4 µm Reprosil C18 resin (Dr. Maisch GmbH, Ammerbuch, Germany) in-house packed picotip emitter tip (diameter 100 µm, 15 cm from New Objectives, Littleton, MA, USA) using a gradient from mobile phase A (4% acetonitrile, 0.1% formic acid) to 50% mobile phase B (99% acetonitrile, 0.1% formic acid) for 30 min with a flow rate 400 nl/min and washout with 99% B for 5 min.

MS data were recorded by means of data-dependent acquisition. The full MS scan range was 300 to 2000 *m*/*z* with a resolution of 70,000, and an automatic gain control (AGC) value of 3 × 10^6^ total ion counts with a maximal ion injection time of 160 ms. Only higher charged ions (2+) were selected for MS/MS scans with a resolution of 17,500, an isolation window of 2 *m*/*z*, and an automatic gain control value set to 10^5^ ions with a maximal ion injection time of 150 ms. MS1 data were acquired in profile mode.

MS data were analyzed by means of MaxQuant (v1.6.1.0) using default settings. Proteins were identified using the proteome database UniProtKB with 10,657 entries, released in 8/2018. The enzyme specificity was set to trypsin. Acetylation (+42.01) at the N-terminus and oxidation of methionine (+15.99) were selected as variable modifications and carbamidomethylation (+57.02) as a fixed modification on cysteines. The false discovery rate (FDR) for the identification protein and peptides was 1%.

Label-free quantification values were obtained from at least one identified peptide. Identifications from a reverse decoy database, based on site and known contaminants, were excluded. The data were further bioinformatically analyzed using Perseus 1.6.1.3. and Microsoft Excel (for MacOS, version 16.16.26). For quantification, the proteins were quality filtered according to a minimum of 3 valid values in one group (*N* = 3). All missing values from this reduced matrix were replaced by the background value representing half of the lowest value in the dataset. For statistical comparisons, Student’s *t*-tests and permutation-based FDR were used. The mass spectrometry proteomics data have been deposited at the ProteomeXchange Consortium via the PRIDE [67] partner repository with the dataset identifier PXD048640. 

### 2.4. Mouse Cerebellar Metabolome

For the quantification of metabolic compounds, 8 WT and 8 CLPP-null sex-matched (50% males, 50% females) 12-month-old mouse cerebella were snap-frozen in liquid nitrogen, stored at −80 °C, and shipped on dry ice to the company Metabolomic Discoveries (Potsdam, Germany). Targeted profiling with unambiguous characterization and relative quantification by means of LC–tandem mass spectrometry was performed on a Shimadzu (Kyoto, Japan) triple quadrupole LCMS-8050 equipped with an electrospray ionization (ESI) source and operated in multiple reaction mode (MRM). For statistical evaluation, the raw data as peak abundances normalized to internal standards and, if necessary, protein content were detailed, complemented by means and standard deviation values, as well as a differential analysis sheet with ANOVA across all groups as a global *p*-value, an adjusted global *p*-value, a local *p*-value from pairwise *t*-tests, ratios of the group means as a log_2_ value, and absolute fold changes.

### 2.5. Mouse Metabolic Validation Study

The whole cerebella from 10 WT and 10 CLPP-null 12-month-old sex-matched mice (40% males, 60% females) were snap-frozen in liquid nitrogen and stored at −80 °C until further processing. Metabolite extraction and tandem LC-MS/MS measurements were performed as previously reported by our group [68]. In brief, methyl-tert-butyl ester (MTBE, Sigma-Aldrich, St. Louis, MO, USA), methanol, ammonium acetate, and water were used for metabolite extraction. The subsequent separation was performed on an LC instrument (1290 series UHPLC; Agilent, Santa Clara, CA, USA), coupled online to a triple quadrupole hybrid ion trap mass spectrometer QTrap 6500 (Sciex, Foster City, CA, USA), as reported previously [69]. The metabolomics data were deposited in the publicly available repository PeptideAtlas with the identifier PASS05856 and can be downloaded at http://www.peptideatlas.org/PASS/PASS05856 (last accessed on 17 January 2024).

The metabolite identification was based on three criteria: (i) the correct retention time, (ii) up to three MRMs, and (iii) a matching MRM ion ratio of tuned pure metabolites as a reference. Relative quantification was performed using MultiQuantTM software v.2.1.1 (Sciex, Foster City, CA, USA), and all peaks were reviewed manually. Only the average peak area of the first transition was used for calculations. Normalization was based on the protein concentration of the samples and subsequently by internal standards.

### 2.6. Quantitative Immunoblots

Cerebellar tissues of 3-month-old male mice (3 WT and 3 CLPP-null) were collected and frozen at −80 °C. Protein isolation and immunoblotting were performed as described earlier [25]. The antibodies used were CLPP (Proteintech, Rosemont, IL, USA), CLPX (NSJ Bioreagents, San Diego, CA, USA), and beta-actin (Sigma Aldrich, St. Louis, MO, USA). The statistical analyses were performed using GraphPad Prism (Version 8).

### 2.7. Statistical Analyses

For statistical analyses, Microsoft Excel was used. Volcano plots were generated with GraphPad Prism, Version 10.1.1, for Windows (Boston, MA, USA).

## 3. Results

### 3.1. Re-Analysis of CLPP Mutation Effects on the P. anserina Metabolome Reveals CLPX as Only CLPP Substrate Associated with PLP, and Highlights OAT among the CLPP Interactors That Could Relate to Prominent Changes in Arginine and Ornithine Levels

For the purpose of the present study into the PLP-dependent metabolism of amino acids, it is first important to reconsider the previous trap-assay study in *P. anserina* [64]. Among the 47 putative CLPP-interacting proteins identified [64], 6 are known for their association with PLP, namely, the ornithine delta-aminotransferase OAT, the aspartate aminotransferase GOT2, the branched-chain amino acid aminotransferase BCAT2, the glycine decarboxylase GLDC, the serine hydroxymethyltransferase SHMT2, and the cysteine desulfurase NFS1.

Among the 19 potential CLPP degradation substrates identified in this study [64], only CLPX associates with PLP, while most other candidate CLPP cleavage targets have cofactors like lipoate, FeS clusters, thiamine diphosphate, or FAD. Therefore, excess CLPX in CLPP-mutant organisms might also modulate other PLP-associated enzymes beyond ALAS, and the binding of other cofactors might be relevant for CLPP cleavage.

**Figure 2:**
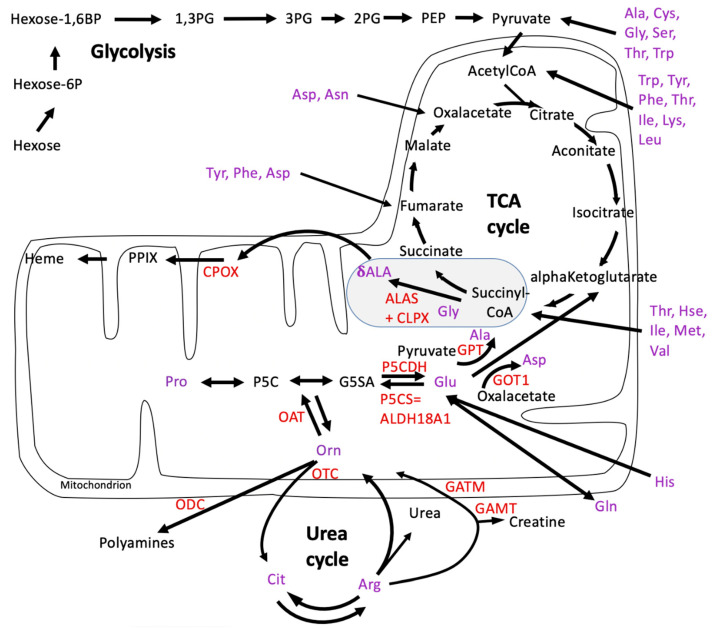
Breakdown of amino acids for consumption in the mitochondrial TCA cycle, and extra-mitochondrial metabolite flux from urea cycle and glycolysis towards heme biosynthesis in wild-type cells. The scheme was modified from the biochemistry textbook Stryer and from [71]. The well-established Shemin pathway of heme biosynthesis is highlighted in an ellipse with a gray background. Enzymes are shown in red letters: ALAS = delta-aminolevulinic acid synthase; CLPX = caseinolytic mitochondrial matrix peptidase chaperone subunit X; CPOX = coproporphyrinogen oxidase; GAMT = guanidinoacetate N-methyltransferase; GATM = glycine amidinotransferase; GOT1 = glutamic-oxaloacetic transaminase 1; GPT = glutamic-pyruvic transaminase; OAT = ornithine delta-aminotransferase; ODC = ornithine decarboxylase; OTC = ornithine transcarbamylase; P5CDH = pyrroline-5-carboxylate dehydrogenase; P5CS = pyrroline-5-carboxylate synthetase = ALDH18A1 = aldehyde dehydrogenase 18 family member A1. Amino acids are shown in purple letters: Ala = alanine; δALA = delta-aminolevulinic acid; Arg = arginine; Asn = asparagine; Asp = aspartate; Cit = citrulline; Cys = cysteine; Gln = glutamine; Glu = glutamine; Gly = glycine; His = histidine; Hse = homoserine; Ile = isoleucine; Leu = leucine; Lys = lysine; Met = methionine; Orn = ornithine; Phe = phenylalanine; Pro = proline; Ser = serine; Thr = threonine; Trp = tryptophan; Tyr = tyrosine; Val = valine. Other metabolites are shown in black letters: CoA = coenzyme A; Hexose-6P = hexose-6-phosphate; Hexose-1,6BP = hexose-1,6-bisphosphate; 1,3PG = 1,3-bisphospho-glycerate; 2PG = 2-phosphoglycerate; 3PG = 3-phosphoglycerate; PEP = phosphoenolpyruvate; PPIX = protoporphyrinogen IX; TCA = tricarboxylic acid.

Next, it is important to reflect on the metabolic profile of CLPP-null *P. anserina* during aging. In contrast to the progressive accumulations of phosphoenolpyruvate and most amino acids from day 5 to 20 in the lifespan, pyruvate stood out with low levels that increased over time, and ornithine appeared prominent with its low levels at day 5 that decreased more over time [63]. The metabolite ornithine is in flux equilibrium (i) via the mitochondrial matrix enzyme OAT with glutamate-5-semialdehyde (GSA or better G5SA) and Δ-1-pyrroline-5-carboxylate (P5C) as precursors of glutamate or proline, respectively (see Figure 2); (ii) via the mitochondrial matrix enzyme ornithine transcarbamylase (OTC) with citrulline as a precursor of arginine; and (iii) via the cytosolic enzyme ornithine decarboxylase (ODC or ODC1) with the polyamine putrescine as a precursor of spermidine/hypusine or spermine (see Figure 2). It was previously shown in plants that OAT is crucial for ornithine generated by arginine breakdown in the urea cycle, but it is not necessary for proline biosynthesis [70], so its primary role is the generation of G5SA. 

**Figure 3:**
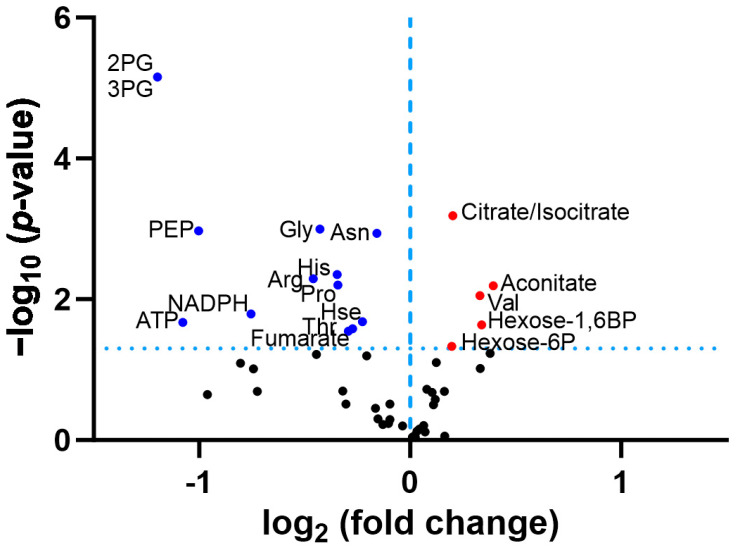
Volcano plot analysis of *P. anserina* CLPP-null metabolome data at day 5 of culture, previously reported in [63]. The significance threshold is represented as horizontal dotted line. Blue dots indicate significant downregulations, red dots indicate significant upregulations. Arg = arginine; Asn = asparagine; ATP = adenosine triphosphate; Gly = glycine; His = histidine; Hexose-6P = hexose-6-phosphate; Hexose-1,6BP = hexose-1,6-bisphosphate; Hse = homoserine; NADPH = nicotinamide adenine dinucleotide phosphate; 2PG = 2-phosphoglycerate; 3PG = 3-phosphoglycerate; PEP = phosphoenolpyruvate; Pro = proline; Thr = threonine; Val = valine.

A re-analysis of the original data in the published *P. anserina* CLPP-null metabolome profile at day 5 of culture (Table S1) revealed that the ornithine decrease was not significant, due to an extremely high statistical outlier among the wild-type values, with the corrected fold change at 0.79 (*p* = 0.14). Among all the amino acids, arginine showed the strongest reduction in fold-change (FC = 0.73; *p* = 0.005), followed by glycine (FC = 0.74; *p* = 0.001), histidine (FC = 0.74; *p* = 0.004), and proline (FC = 0.79; *p* = 0.006), as shown in Figure 3. While the Gly decrease can be explained by the increased δALA production, the causes of the Arg/His/Pro decreases are still unknown. The low levels of 2PG/3PG/PEP, together with the accumulation of hexose-6-phosphate and hexose-1,6-bisphosphate, suggest an altered glycolytic flux. In the TCA cycle, the lower levels of fumarate contrasted with the elevated levels of citrate/isocitrate/aconitate. All findings can be explained by maximized heme synthesis within the following scenario where the decreased fumarate and Gly levels are due to increased sequestration of succinyl-CoA and glycine for δALA biosynthesis, reflecting low TCA cycle activity with resulting bioenergetics deficits (low NADPH and ATP). Cellular efforts to replenish succinyl-CoA would catabolize Thr and Hse and would generate alpha-ketoglutarate by way of the breakdown of Arg/His/Pro (see Figure 2). Other signals would accelerate glycolytic breakdown to provide acetyl-CoA, in parallel to Asn catabolism to oxaloacetate, for maximized production of citrate/aconitate/isocitrate, which accumulate before the step of alpha-ketoglutarate/succinyl-CoA conversion to δALA. 

**Figure 4:**
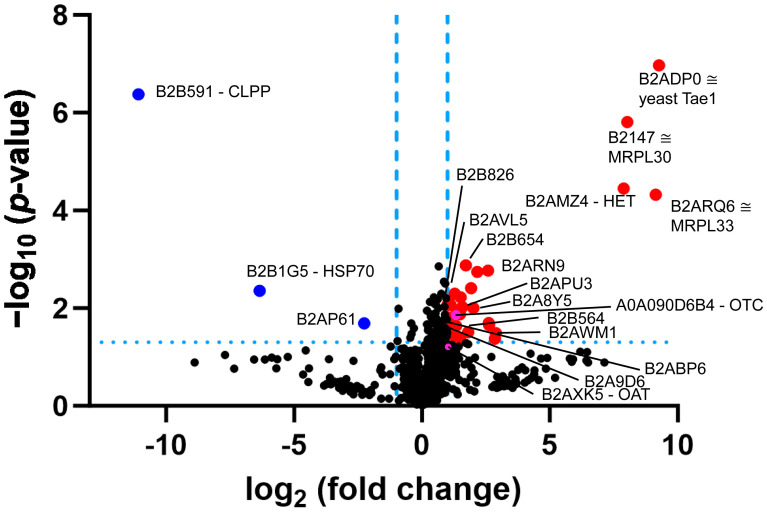
Volcano plot analysis of *P. anserina* CLPP-null proteome profile after 8 days of culture. Blue dots indicate significant (*p* < 0.05) downregulations, and red dots indicate significant upregulations. Symbols beyond the dotted threshold line for significance have maximal stringency and credibility. HSP70 = heat shock protein 70 kDa; Tae1 = yeast alpha N-terminal protein methyltransferase 1; MRPL30 = mitoribosomal large subunit protein uL30m; MRPL33 = mitoribosomal large subunit proteins bL33; HET = heterokaryon incompatibility protein; OAT = ornithine delta-aminotransferase; OTC = ornithine transcarbamylase. To distinguish the dysregulation of the last two proteins from the surrounding cloud, their positions in the volcano plot are highlighted as purple dots.

New proteome profiling data of Δ*PaClpP* obtained by means of label-free mass spectrometry quantified the 1308 most abundant proteins (Table S2). The findings confirmed the absence of CLPP (UniProt-ID B2B591) but failed to detect the known CLPX homolog (B2B254 or A0A090CGT2), probably due to technical issues. The data revealed elevated levels for the homologs of mitochondrial OTC (A0A090CVB0, 2.04-fold, *p* = 0.02) and mitochondrial OAT (B2AXK5, 1.75-fold, *p* = 0.09). In contrast, the homologs of GLDC, GOT2, BCAT2, NFS1, and SHMT2, which are putative CLPP interactors, were not found among these abundant dysregulated proteins. An increased OAT abundance has not been observed in ClpP-null bacteria, but it is consistent in the eukaryotes *P. anserina*, *Mus musculus*, and *Homo sapiens* [7,72], so it might be a consequence of the heme biosynthesis switch from the Beale C5 pathway in bacteria to the Shemin C4 pathway in eukaryotes. Interestingly, the volcano plot analysis of this proteome profile (Figure 4) reveals four massive upregulations in response to the absence of CLPP: an S-adenosyl methionine (SAM)-dependent O-methyltransferase (B2ADP0, homologous to yeast Tae1), homologs of the mitoribosomal large subunit proteins uL30m (B2B147, ferredoxin-like) and bL33 (B2ARQ6, zinc-binding), and a heterokaryon incompatibility protein (B2AMZ4). 

The dysregulation of SAM-dependent methyltransferases is substantiated further by the significant upregulation of B2B823 and B2AQK9 (Figure 4 and green background in Table S2). If CLPX is involved not only in PLP/vitamin-B6 binding to enzymes but also in cobalamin/vitamin-B12 binding to enzymes, then this general accumulation of several SAM-dependent enzymes might be due to altered recycling of SAM by methionine synthase [15]. A SAM-dependent methyltransferase known in the mammalian metabolism of ornithine/arginine is GAMT (guanidinoacetate N-methyltransferase) (Figure 2). More importantly, SAM-dependent methyltransferases mediate the maturation of tRNAs and ribosomes, as well as translation in mitochondria [73]. In particular, they modulate a late-stage assembly checkpoint of the mitoribosomal large subunit (LSU) [74]. The massive accumulation of two mitoribosomal LSU subunits in CLPP-null *P. anserina* is further emphasized by milder but significant accumulations of the proteins B2AWM1, B2ARN9, B2A8Y5, B2B564, B2B654, B2APU3, B2B826, B2AVL5, B2ABP6, and B2A9D6 (Figure 4 and yellow background in Table S2), which are components of the mitoribosomal LSU and the translation elongation pathway. These findings are consistent with the selective impact of the absence of CLPP on two mitoribosomal LSU intermediate assemblies in mouse tissues, which was recently observed [25]. Nothing is known about the cellular functions of the heterokaryon incompatibility protein B2AMZ4. 

Further pathway dysregulations in Table S2 include (i) the respiratory chain complexes I/IV with effects on B2B1C3, B2B7U7, A0A090CEV3, B2AR53, B2AWS7, B2AT34, B2AR06, B2B5R1, P20682, and B2AYP8 (orange background); (ii) the molecular chaperones with effects on A0A090CCG8 and B2B1G5 (lilac background); (iii) the acetyl-CoA and acyl-CoA synthases A0A090D6B4 and B2B6I3 (light red background); and (iv) the putative prolyl-3-hydroxylase-2 A0A090CDR3 that would modulate the UPR^mt^ via ATF4 (blue background) [75].

Overall, this proteome profile of CLPP-null *P. anserina* showed selective dysregulation of the mitoribosomal LSU and respiratory complex I/IV factors, as well as molecular chaperones, just like the CLPP-null mouse proteome profiles [25,47]. 

### 3.2. Serine and Arginine Show the Strongest Reductions among Cognate Amino Acid Levels in CLPP-Null Mouse Cerebellum

**Figure 5:**
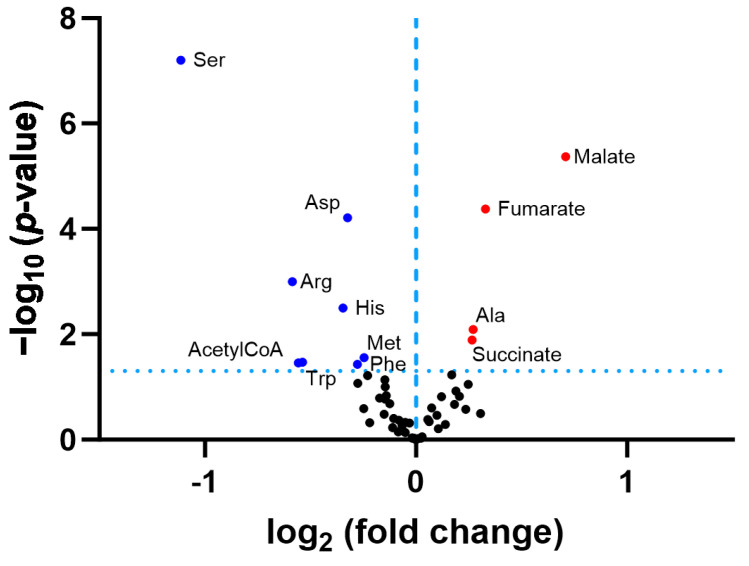
Volcano plot analysis of adult *CLPP*-null cerebellum metabolome. The significance threshold is represented as a dotted line. Blue dots indicate significant (*p* < 0.05) downregulations, and red dots indicate significant upregulations.

To further elucidate by metabolomics whether classical amino acid levels and their catabolism via gluconeogenesis or ketogenesis show similar changes in mice as in *P. anserina*, a first-tier basal metabolic screen was performed in CLPP-null mouse cerebellum, a tissue preferentially affected by the progressive ataxia in PRLTS3 patients. We chose this central nervous tissue also because the blood–brain barrier stabilizes amino acid homeostasis in the nervous system and counteracts the variations due to periodic food uptake. The absence of CLPP and the excess abundance of CLPX (12.24-fold increase, *p* = 0.0003) in the cerebella was verified by means of quantitative immunoblots (Figure S1). This first-tier survey was performed by a commercial service and documented significantly lowered levels for many amino acids (Ser, Arg, His, Trp, Asp, Phe, Met) including arginine, while ornithine levels were too low for detection using this approach (Figure 5 and Table S3). The prominent reduction in Ser levels is probably correlated with the consistent accumulation of SHMT2 protein in mammalian CLPP-null cells (see Figure 2 in [47]), which consumes Ser to resupply the Gly levels that are depleted by δALA biosynthesis. In parallel, SHMT2 transfers one carbon to 5,10-methylenetetrahydrofolate (5,10-MTHF), a key metabolite for the folate and methionine cycles, which generate S-adenosyl-methionine as a cofactor for methyltransferases. Indeed, the mitochondrial bifunctional methylenetetrahydrofolate dehydrogenase/cyclohydrolase MTHFD2, the enzyme immediately downstream of the Ser-Gly conversion, showed a 3-fold increase in abundance in CLPP-null mouse brain proteome profiles (see Table S3 in [47]).

The only amino acid with abnormally high levels was alanine, which is generated from pyruvate via transamination when the enzyme GPT releases the amino group from glutamate to generate alpha-ketoglutarate (see Figure 2), which is a source of succinyl-CoA for subsequent δALA biosynthesis. Again, the TCA cycle activity appeared affected by the CLPP-null mutation given that the intermediate metabolites fumarate, succinate, and malate had accumulated and acetyl-CoA (a substrate of consumption by the TCA cycle) showed low levels (Figure 5 and Table S3). Overall, the data indicated that low levels of many amino acids contrast with high levels of many keto-acids, so in mouse, the PLP-dependent transaminations may be affected subtly in a general manner. 

### 3.3. Confirmatory Survey with Selected Non-Cognate Amino Acids in CLPP-Null Mouse Cerebellum

To re-assess the effects of CLPP deficiency on amino acid metabolism with additional samples in an independent academic lab, and to elucidate how non-proteinogenic amino acids are affected in CLPP-null cerebellum, a second-tier metabolomic validation study was conducted. This approach confirmed significantly low Arg and Ser levels, as well as high Ala amounts after multiple testing correction, and low Gln, acetyl-Gln, and Asp, with high Pro levels of nominal significance (Table S4). These findings are compatible with a scenario where the increased δALA biosynthesis from Gly and succinyl-CoA is compensated by Ser and alpha-ketoglutarate. The production of the latter reduces Gln/acetyl-Gln stores while increasing Ala. In addition, the accelerated breakdown of Arg leads to more Pro, and Asp is consumed to replenish fumarate (see Figure 2). 

**Figure 6:**
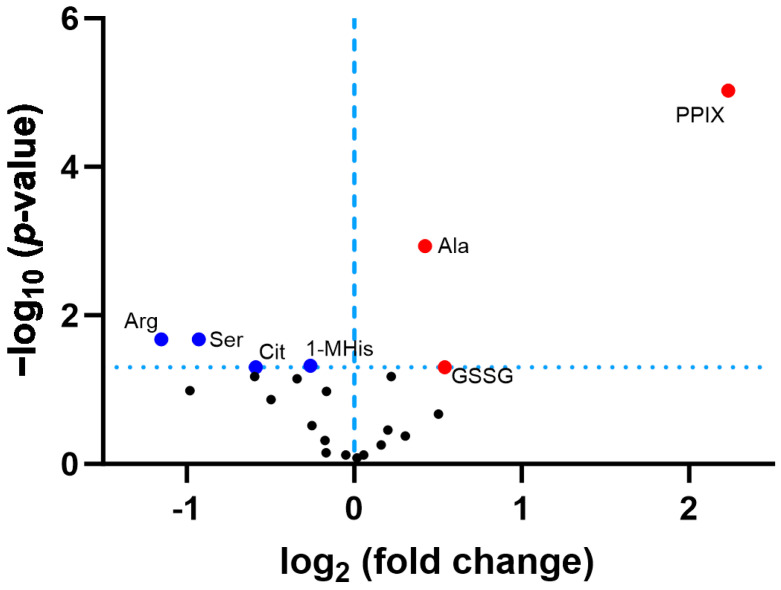
Volcano plot of cognate and selected non-cognate amino acids and derivatives in adult CLPP-null cerebellum. The significance threshold is represented as a dotted line. Blue dots indicate significant downregulations, and red dots indicate significant upregulations. GSSG = oxidized glutathione; 1-MHis = 1-methylhistamine; PPIX = protoporphyrinogen IX.

In addition, reduced steady-state levels were found for the non-canonical amino acid derivatives citrulline (Cit, and its derivatives Orn and Arg, share amino groups at the alpha- and delta-carbon, see Figure 1) (FC = 0.67; *p* = 0.02) and 1-methylhistamine (1-MHis, the main catabolite of histamine, which is derived from histidine and has a second amino group attached to the delta-carbon) (FC = 0.83; *p* = 0.01). Histidine was not studied in this analysis. In contrast, excess quantities were documented for oxidized glutathione (GSSG, with a delta-amino-group) (FC = 1.46; *p* = 0.02) and the (δALA-derived) heme precursor protoporphyrin IX (PPIX) (FC = 4.70; *p* = 4.1 × 10^−7^) (Figure 6). In comparison to the 30% accumulation of alanine (Ala, FC = 1.34; *p* = 0.0001), the accumulation of the δALA-derived PPIX was much higher (370%), probably a simple reflection of the fact that there is much more Ala than δALA in any cell. Compared with the 47% reduction in serine (FC = 0.53; *p* = 0.004), the 55% decrease in arginine (Arg, FC = 0.45; *p* = 0.004) was stronger in this experiment, a novel observation that may suggest that δALA biosynthesis is fueled more by Arg/Orn than by Gly/Ser in the CLPP-null mouse cerebellum. Furthermore, non-proteinogenic Cit showed a stronger decrease than all other classical amino acids (Figure 6). In contrast, the basal polyamine putrescine was not elevated (Table S4). Overall, these mouse data suggest that the absence of CLPP with excess CLPX may influence PLP-dependent transamination more strongly at delta-carbon positions than at alpha-carbons. 

## 4. Discussion

### 4.1. Absence of CLPP Affects Amino Acid Metabsolism

The main novel finding of this study in both mouse cerebellar tissue and young *P. anserina* cultures is that the absence of CLPP leads to consistent and particularly strong reductions in Arg and its related compounds Cit and His, which share a C5 core and (attached at the delta-carbon position) a guanidino group that is metabolized to keto-acid or circular form. All three compounds can be converted to the metabolite alpha-ketoglutarate which is the substrate for succinyl-CoA generation within the TCA cycle and are thus presumably channeled into the excess biosynthesis of δALA. 

We have to consider that the absence of CLPP is known to result in a several-fold excess of CLPX, from all bacteria analyzed at present, until complex eukaryotes such as mouse and human [7]. Although the levels of PaCLPX escaped detection in the current proteome profiles, PaCLPX was identified by mass spectrometry after enrichment in previously published CLPP substrate trapping experiments [64]. Increased CLPX abundance leads to heme biosynthesis activation via PLP binding to ALAS, which utilizes succinyl-CoA and glycine. Indeed, if succinyl-CoA is not broken down into succinate/CoA/GTP within the TCA cycle, but instead used to build δALA in the heme biosynthesis process, then the GTP reduction to 21% observed in Table S1 can be explained with ease. However, such a chronic sequestration of a metabolite from the TCA cycle would hamper the cellular bioenergetics, and indeed the significant reduction in ATP levels in the *P. anserina* metabolome data reflects a mitochondrial bioenergetic deficit. The C4 compound succinyl-CoA could be replenished more directly by the breakdown of amino acids with a C4 core chain, namely Thr, Hse, Val, and Met (see Figure 2), but this option is only partially supported by our observations. As a stronger contribution, an apparently more indirect replenishment within the TCA cycle from the C5-chain compound alpha-ketoglutarate, and externally via Glu by the breakdown of C5-chain amino acids Orn, Arg, and Cit is apparent (see Figure 1 and Figure 2). However, the levels of Glu/Gln are not strongly altered, presumably because they are replenished from His. It is important to note here that an accumulation of the P5CS/ALDH18A1 enzyme was consistently documented in mammalian CLPP-null proteome profiles (2-fold in brain, see Table S3 in [47]; 3-fold in testis at P21, see Table S1 in [54]), indicating increased activity producing G5SA from Glu as well as from Orn, Arg, and Cit. Therefore, it is conceivable that the increased G5SA generation can be channeled directly into δALA biosynthesis without detour conversions to Glu/alpha-ketoglutarate/succinyl-CoA. This might occur within a single step, if OAT after PLP activation by excessive CLPX abundance undergoes a gain of a GSA 1,5-aminomutase function. To convert G5SA to δALA, an enzyme would have to bind to the carboxy group and then reposition the amino group from the alpha-carbon to the delta-carbon, and the keto group from the delta-carbon to the gamma-carbon (see Figure 1). In view of the sequence homology between the bacterial GSA 2,1-aminomutase HemL and mitochondrial OAT, together with their structural and mechanistic similarities [76], this seems conceivable. Thus, a remainder of the archaeal C5 pathway of ALA biosynthesis might be activated to coexist with the eukaryotic C4 pathway in cells with maximized heme biosynthesis due to an excess of CLPX. Of course, such a direct conversion of G5SA to δALA via OAT together with the more indirect conversion of G5SA via Glu/alpha-ketoglutarate/succinyl-CoA to δALA would both contribute Arg to heme biosynthesis, while elevating the alanine levels. This scenario would explain the observations of the five-fold increased levels of PPIX with the concomitant two-fold reduced levels of Cit and Arg (and Orn?) via the C5 pathway, plus the two-fold decrease in Gly or Ser via the C4 pathway, providing a tentative mechanistic explanation of several of the metabolome findings.

These novel metabolomic findings can be explained by the accumulation of excess ornithine delta-aminotransferase (OAT), shown in Table S2 for *P. anserina*, which was previously also documented for mouse tissues [25]. In *P. anserina*, it is known that OAT is an interactor of overexpressed WT CLPP protein [64], and previous work in mouse testis tissue confirmed protein–protein interactions between endogenous OAT and CLPX [25]. The quaternary structure of OAT is a homohexameric ring, where each subunit is activated by association with PLP. Thus, it is likely that CLPX, as a monomer or homoheptameric ring, unfolds the OAT ring to enable its activation by PLP. Overactive OAT would metabolize Arg-derived Orn, acting either as a delta-transaminase to produce G5SA (see Figure 1) for subsequent conversion to Glu and the C4 compound succinyl-CoA, or acting first as a delta-transaminase and then also as a GSA 1,5-aminomutase to produce δALA directly (see Figure 1). Orn (even more than Cit) is a transient intermediate and is usually below detection limits in mammals. However, it plays a crucial role at the crossroads between amino acid metabolism, polyamine biosynthesis, urea excretion, and creatine bioenergetics (see Figure 2); therefore, the regulation of its processing by OAT is probably decisive. 

Thus, the excess CLPX in CLPP-null organisms could overactivate ALAS and OAT, jointly causing lower arginine levels. It is important to note that arginine contains four amino groups per molecule, so in mammals, it represents the main nitrogen storage reserve for growth [77]. Body length and muscle mass are critically dependent on the availability of nitrogen, which is taken up mainly in the form of amino acids within proteins [78]. The levels of arginine are, therefore, a decisive signal in the pituitary signaling pathway to secrete growth hormone (GH) [79]. This is also the reason why arginine is used routinely by body builders as a diet “pump-supplement” [80]. The reduction in arginine to 47% in CLPP-null mouse tissues (Table S4) is, therefore, an excellent explanation for their decreased body weight to around 50% [12], and for the small body size of PRLTS3 patients (below the third percentile) [44]. It is relevant to know that dietary supplementation with L-arginine was successfully used to rescue the growth deficits of a patient with mitochondrial Barth syndrome where unresponsiveness to GH was observed [81]. Thus, we propose that supplementation with excess arginine in the diet of PRLTS3 patients will partially prevent and revert their growth deficit. In contrast to this mammalian phenotype, growth is not affected in the *P. anserina* CLPP-null mutant [62], probably because nitrogen is readily available in cells (e.g., in the chitin of the cell wall) and in nature, on the substrate (herbivorous dung) on which this fungus grows. The nitrogen storage role of arginine is, therefore, not critical in this fungus.

Of course, the question arises if (i) the increased heme biosynthesis, (ii) TCA cycle impairment due to succinyl-CoA depletion with a consequent reduction in GTP and acetyl-CoA, and (iii) combined Arg/Cit/His decreases are also relevant for the impact of CLPP antagonist drugs on the growth of bacteria and cancer cells. It also remains to be explored if the effect of CLPXP-modulated, PLP-dependent amino acid metabolism has an overall impact on the urea cycle and organismal nitrogen utilization versus release.

**Figure 7:**
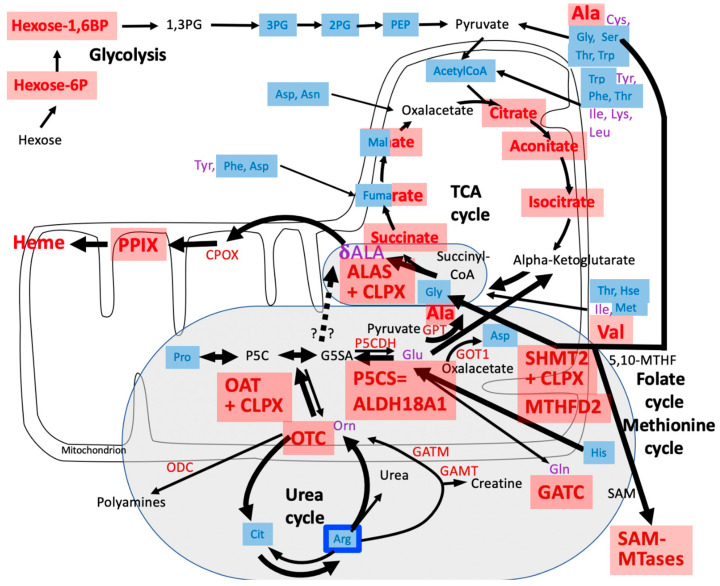
Summary of *P. anserina* culture and mouse cerebellum observations, how the breakdown of amino acids for consumption in the mitochondrial TCA cycle, and extra-mitochondrial metabolite flux from urea cycle and glycolysis towards heme biosynthesis is changed in CLPP-null cells, compared to the WT scenario in Figure 2. As in Figure 2, the Shemin pathway (primarily responsible for heme biosynthesis) is highlighted in an ellipse with a gray background. In Figure 6, an additional gray ellipse emphasizes the recruitment of further metabolites when overactivated heme biosynthesis occurs in the absence of CLPP, when there is excess CLPX. Whether the sequence homology and structural similarity between OAT and GSA aminomutase reflects an ancient processing of ornithine as a C5 substrate for δALA biosynthesis remains to be tested further and is highlighted by question marks. Broader arrows suggest an increased metabolite flux in CLPP-null eukaryotes. Blue background and smaller font indicate downregulation, red background and larger font indicate upregulations; bicolor background reflects different regulations in *P. anserina* versus mouse. The consistent arginine reduction is highlighted by a blue frame. Enzymes are shown in red letters: ALAS = delta-aminolevulinic acid synthase; CLPX = caseinolytic mitochondrial matrix peptidase chaperone subunit X; CPOX = coproporphyrinogen oxidase; GAMT = guanidinoacetate N-methyltransferase; GATC = glutamyl-tRNA amidotransferase subunit C in mitochondria; GATM = glycine amidinotransferase; GOT1 = glutamic-oxaloacetic transaminase 1; GPT = glutamic-pyruvic transaminase; MTHFD2 = bifunctional methylenetetrahydrofolate dehydrogenase in mitochondria; OAT = ornithine delta-aminotransferase; ODC = ornithine decarboxylase; OTC = ornithine transcarbamylase; P5CDH = pyrroline-5-carboxylate dehydrogenase; P5CS = pyrroline-5-carboxylate synthetase = ALDH18A1 = aldehyde dehydrogenase 18 family member A1; SAM MTases = S-adenosyl methionine-dependent methyltransferases; SHMT2 = serine hydroxymethyltransferase 2 in mitochondria. Amino acids are shown in purple letters: Ala = alanine; δALA = delta-aminolevulinic acid; Arg = arginine; Asn = asparagine; Asp = aspartate; Cit = citrulline; Cys = cysteine; Gln = glutamine; Glu = glutamine; Gly = glycine; His = histidine; Hse = homoserine; Ile = isoleucine; Leu = leucine; Lys = lysine; Met = methionine; Orn = ornithine; Phe = phenylalanine; Pro = proline; Ser = serine; Thr = threonine; Trp = tryptophan; Tyr = tyrosine; Val = valine. Other metabolites are shown in black letters: 5,10-MTHF = 5,10-methylene-tetrahydrofolate; CoA = coenzyme A; Hexose-6P = hexose-6-phosphate; Hexose-1,6BP = hexose-1,6-bisphosphate; 1,3PG = 1,3-bisphospho-glycerate; 2PG = 2-phosphoglycerate; 3PG = 3-phosphoglycerate; PEP = phosphoenolpyruvate; PPIX = protoporphyrinogen IX; SAM = S-adenosyl methionine; TCA = tricarboxylic acid.

The deficit in glycine in the metabolic profile of CLPP-null *P. anserina* might simply reflect the excessive δALA synthesis from Gly and the C4 substrate succinyl-CoA (see summary scheme in Figure 7 below). In mitochondria, Gly is degraded to amino groups and CO_2_ by the PLP-dependent enzyme GLDC and is converted to Ser by the PLP-dependent enzyme SHMT2, both of which were identified as CLPP interactors [64]. Thus, the prominent Ser reduction in CLPP-null mouse cerebellum, together with the elevated abundance of SHMT2 and MTHFD2, might reflect an excessive activity of PLP-activated SHMT2.

In agreement with the concept that the archaeal/bacterial C5 pathway of δALA biosynthesis is not conserved in mammals because an ortholog of the glutamyl-tRNA reductase HemA does not exist, there was only a statistical trend for a 20% increase for the GltX ortholog EARS2, a mitochondrial glutamyl-tRNA synthase, in the CLPP-null mouse brain (see Table S3 in [47]). However, it is interesting to note that the abundance of the mitochondrial glutaminyl-tRNA synthase component GATC showed an even stronger significant elevation (7.1-fold) than ALAS1 (3.9-fold) in CLPP-null mouse brain proteome profiles (see Table S3 in [47]), suggesting that tRNA-Gln instead of tRNA-Glu might be recruited as a C5 source for heme production in PRLTS3 (see Figure 1).

The reduction in His was less prominent than the reduction in Arg in the three surveys performed but had a higher fold-change than the remaining proteinogenic amino acids. Lower His levels would have an impact on growth [82,83,84,85,86], metal ion chelation (e.g., Fe^2+^ binding to heme via His, which generally serves as ligand within metalloproteins), heavy metal stress sensing (histidine kinases), scavenging of reactive oxygen and nitrogen species, and histaminergic signaling [87]. The degradation of His to Glu is not modulated by PLP, so its decreased levels can be only explained by the increased flux to replenish alpha-ketoglutarate and succinyl-CoA in the TCA cycle. However, PLP dependence exists for the conversion of His to histamine by the enzyme histidine decarboxylase. The deficiency in histamine is supported by the low levels of 1-methylhistamine in the metabolome profile of the CLPP-null cerebellum. Histamine acts as a signaling molecule and is stored in mast cells below body surfaces [88]. A lack of histamine would reduce the itchy feelings and inflammatory responses upon bacterial infection of the skin. The His deficit and the likely histamine deficit, therefore, explain the resistance to ulcerative dermatitis that was observed for CLPP-null mice with a C57BL/6 genetic background [12,59].

### 4.2. Absence of CLPP Affects Mitoribosomal LSU and Its rRNA/tRNA^Val/Phe^

A second novel finding of this study in the *P. anserina* CLPP-null proteome profile is the strong selective accumulation of two components of the mitoribosomal LSU. Similarly, in CLPP-null mice, CLPX was shown to have a selective effect on mitoribosomal LSU components [25]. Both in *P. anserina* and in mouse CLPP-null organisms, the affected proteins are constituents of the LSU central protuberance, which contains the 5S rRNA or tRNA^Val/Phe^ which associates with LSU 16S rRNA [89]. 

In the *P. anserina* CLPP-null proteome profile, two LSU proteins showed a massive upregulation. B2B147 has a ferredoxin-like β-α-β-α-β-fold core structure, and B2ARQ6 has a zinc-binding domain, so both factors potentially associate with heavy metals, and recent mouse analyses documented several heavy metals accumulating in CLPP-null tissues [25].

Additional ribonucleoproteins also significantly accumulated with a modest fold change, but they appear to represent a very selective pattern acting on LSU components including the following:

B2AVL5 (L31 family) is homologous to a bacterial ribosomal LSU component that associates with 5S rRNA and is lost from mitoribosomes in mammals [89].

B2ARN9 (mL41 family) is a mitochondria-specific protein that compensates for lost rRNA in the mitoribosomal LSU [89].

B2A8Y5 (mL53 family) is part of the glutaredoxin superfamily and is a mitochondria-specific protein that connects the L7/L12 stalk to the central protuberance of the mitoribosome next to uL30 [89].

B2B826 (ribosomal protein P1/P2) is localized in the L7/L12 stalk and has important roles in the elongation step of protein synthesis, as was shown for cytosolic ribosomes [90].

B2ABP6 is the synthetase for tRNA^Val^, which replaced 5S rRNA in the LSU central protuberance during eukaryotic evolution [91]. 

B2AWM1 (homolog of eIF6) is required for LSU biogenesis of 60S subunits; its disassociation permits binding to the SSU and formation of the translation initiation complex [92]. Its binding occurs near the L7/L12 stalk at uL3/uL11/uL14/uL16/L23/L24 in competition with translation elongation factor G, mouse GFM1/GFM2 [93,94,95]. It belongs to the pentein superfamily of peptidylarginine deiminases (PADI), along with succinylarginine dehydrolase, dimethylarginine dimethylaminohydrolase, and arginine–glycine amidinotransferase (AGAT/GATM, see Figure 2), and it can be blocked by the protein arginine methyltransferase antagonist drug eIFsixty-4 [96]. While it is unclear if it retains enzymatic activity, it does presumably bind to a peptidylarginine moiety. Therefore, the dysregulation of this factor provides a clear link with the arginine deficit in CLPP-null organisms. 

B2B654 (NOP10 family) is a subunit of the H/ACA ribonucleoprotein complex responsible for the pseudouridylation of rRNA, tRNA, and snoRNA [97,98].

B2APU3 (GAR1 family, metallophosphatase DUF2433 family) is also a subunit of the H/ACA ribonucleoprotein complex responsible for the pseudouridylation of rRNA, tRNA, and snoRNA [97,98].

While all the above factors selectively target the mitoribosomal LSU and its rRNA or tRNA^Val^, the following proteins are thought to act on mRNAs or are poorly understood ribonucleoproteins:

B2A9D6 (small nuclear ribonucleoprotein G) with an Lsm7/SmG-like domain. It assembles with other Sm proteins into a hetero-heptameric ring around the Sm site of the 2,2,7-trimethyl guanosine (m3G)-capped U1, U2, U4, and U5 snRNAs (Sm snRNAs), forming the core of the snRNP particle [99]. The snRNP particle, in turn, assembles with other components onto pre-mRNA to form the spliceosome which is responsible for the excision of introns and the ligation of exons.

B2B564 contains a DNA/RNA-binding SAP domain at the N-terminus.

Thus, the mitoribosomal translation fidelity and elongation problems previously reported in CLPP-null mice do not seem to be only caused by a lack of GTP or a deficiency in hypusine, which results in the accumulation of translation elongation proteins and ribosome recycling proteins, but rather reflect specific alterations to mitoribosomal LSU rRNA/tRNA^Val^ factors. Whether abnormal methylation of rRNA or peptidylarginine plays a role remains unclear. 

What is the link between CLPXP’s delta-amino acid preference and the mitoribosomal translation pathology? There is no established link between PLP and pseudouridylation or SAM-dependent methylation in mitochondrial RNA processing. However, it is conceivable that the generation of polypeptide chains in the ribosome might have a small error rate leading to bond formation between the polypeptide carboxy-terminus at the P-site, and the delta-amino group instead of the alpha-amino group of the subsequent amino acid-tRNA at the A-site. Such misincorporation problems would lead to steric problems in the nascent peptide exit channel of the LSU. It might be a function of CLPX to recognize such bonds, and the crucial role of CLPP to degrade such abnormal polypeptides that would be marked with a C-terminal alanine–threonine-tail before release from the translation machinery. Such translation fidelity issues would arise more frequently during cell stress [100]. As an alternative link, changes to mitochondrial C1 metabolism may lead to abnormal methylation during RNA maturation, triggering the misassembly of RNA with its processing and translation proteins, and thus leading to UPR^mt^.

### 4.3. Absence of CLPP Affects SAM-Dependent Methyltransferases

A third novel finding of this study in the *P. anserina* CLPP-null proteome profile is the massive accumulation of the SAM-dependent O-methyltransferase B2ADP0, and the modest but significant upregulations of the SAM-dependent methyltransferase-7B-like B2B823 and the SAM-dependent O-methyltransferase B2AQK9. The reason for this SAM-centered pathology might be explained by altered SAM regeneration in *P. anserina*. At least in mammals, the SAM cycle depends on the cofactor cobalamin whose archaeal/bacterial biosynthesis depends on δALA availability [15], and the association of cobalamin with the enzyme methionine synthase might be modulated by CLPX similar to the PLP-ALAS association. As an additional or alternative explanation, SAM MTases may have abnormal activity due to the changes in mitochondrial C1 metabolism downstream from the excess SHMT2 and MTHFD2. What would be the downstream consequences of these dysregulations? Within the superfamily of SAM-dependent methyltransferases, the most abundant and best-studied class A is defined by a partial (βα)-6 barrel structure, associates with a FeS-cluster, and acts in the processing of rRNAs and tRNAs [101,102,103,104,105]. Therefore, the strong accumulation of the mitoribosomal proteins B2B147 and B2ARQ6 might be due to abnormal LSU rRNA methylation by B2ADP0, suggesting a massively abnormal pathway in the maturation of the large ribosomal subunit in the CLPP-null *P. anserina* strain. B2ADP0 contains a winged-helix DNA-binding domain, homologous to the arginine repressor, so the dysregulation of this factor could also be related to the ornithine metabolism anomaly.

Interestingly, very close homologs of B2ADP0 (according to the STRING webtool) in the fungus *Aspergillus nidulans* are the enzymes asqN and asqD, which mediate steps in the biosynthesis of aspoquinolone, a terpenoid mycotoxin [106], functioning after a SAM-dependent step catalyzed by the nonribosomal peptide synthetase asqK. The close homologs in the fungus *Neurospora crassa* include sterigmatocystin 8-O-methyltransferase and other enzymes that are also key to the biosynthesis of polyketide mycotoxins [107,108]. These findings raise the possibility that *P. anserina* CLPP and CLPX modulate bonds between non-classical amino acids in the production of cyclic venoms, as well as signaling molecules. In the yeast species *Saccharomyces cerevisiae* and *Schizosaccharomyces pombe*, the closest homolog is Tae1, an alpha-N-methyltransferase that methylates the N-terminal motif [Ala/Pro/Ser]-Pro-Lys of target proteins when the initiator Met has been cleaved. Tae1 modifies several ribosomal proteins [109,110,111]. Therefore, an additional possibility is that CLPXP, via SAM-dependent methylation, modulates the N-terminal processing of target proteins. However, the cytosolic enzymes ASMT, NTMT1, and NTMT2, the closest homologs of B2ADP0 in mice, did not show a significant dysregulation in CLPP-null proteome profiles. Instead, a prominently and consistently elevated abundance was observed for the mitochondrial S-adenosyl-methionine-dependent N-methyltransferase TRMT10C (also known as MRPP1) in several CLPP-null mouse tissues (3.1-fold in brain, see Table S3 from [47]; 2-fold in testis at P17 and P21, see Table S1 from [54]). TRMT10C acts together with two other components of the RNAse-P complex in order to excise tRNAs from the mitochondrial polycistronic transcript and to ensure their correct folding [112,113,114,115]. Its function requires interactions with the RNAse-P endonuclease component PRORP (=MRPP3), where mutations are known to also cause a Perrault syndrome phenotype [49]. Any problems in the maturation of diverse tRNAs due to dysfunction of TRMT10C or PRORP would of course impair the assembly of the mitoribosomal LSU central protuberance around tRNA^Val/Phe^, and even in successfully assembled mitoribosomes, the translation efficiency would be reduced. 

Previous *P. anserina* work has already demonstrated the accumulation of another mitochondrial SAM-dependent methyltransferase (UniProt-ID Q9HGR1) during the aging process [116], and the protective effect of its overexpression on lifespan [117], while its deletion resulted in vulnerability towards metal-triggered oxidative stress [118]. Our current data may correlate the accumulation of SAM-dependent O-methyltransferase B2ADP0 as the main proteome finding with the extended lifespan observed for the CLPP-null strain. It should be mentioned that the superfamily of methyltransferases contains spermidine synthase as its ancient prototype, so it is also possible that the methylation targets a polyamine rather than a ribonucleoprotein complex. Polyamines are crucial for growth, healthy aging, and a normal lifespan [119,120,121,122,123].

### 4.4. Absence of CLPP Affects Molecular Chaperones and the UPR^mt^

A fourth novel finding of this study in the *P. anserina* CLPP-null proteome profile is the modest accumulation of molecular chaperones and a potential UPR^mt^ regulator.

A0A090CCG8 is a BAG domain-containing protein. Bcl2-associated athanogene (BAG) family proteins can interact with the HSP70 proteins and regulate them, exerting an anti-apoptotic effect [124]. Therefore, its accumulation may reflect the UPR^mt^ via molecular chaperone upregulation. However, it is interesting to note that STRING homology searches in *Neurospora crassa*, *Caenorhabditis elegans, Drosophila melanogaster, Danio rerio, Xenopus laevis, Mus musculus*, and *Homo sapiens* found a low similarity between A0A090CCG8 and the lysosomal cobalamin transporter LMBRD1, an interesting observation given the cobalt accumulation documented in CLPP-null mouse tissues [25]. Cobalamin production in archaea/bacteria depends on the availability of corrin rings that are produced from δALA. Cobalamin is an essential cofactor for mitochondrial methylmalonyl-CoA mutase and for methionine synthase in the re-methylation of homocysteine to methionine with concurrent SAM regeneration, which is crucial for all S-adenosylmethionine-dependent methylation reactions [125,126]. Of course, cobalamine binding to mammalian enzymes might also be modulated by CLPX, similar to PLP binding to ALAS and OAT.

B2B1G5 is homologous to DnaK and belongs to the HSP70 family. Its accumulation suggests that the altered unfoldase/disaggregase role of CLPX results in a compensatory upregulation and/or delayed turnover of the HSP70 chaperone system, reflecting cellular efforts to correct misassembled protein complexes.

This is in line with the significant accumulation of A0A090CDR3, a protein with sequence homology to prolyl-3-hydroxylase-2. In human cell cultures, the prolyl hydroxylase domain-containing oxygen sensor PHD3 modulates UPR^mt^ via the oxygen-dependent transcription factor ATF4 [75]. Thus, it is conceivable that the abnormal mitochondrial translation and membrane insertion of respiratory chain core proteins will trigger UPR^mt^ and ATF4 activation together with dysregulation of its upstream prolyl-3-hydroxylase regulator.

In CLPP-null mouse tissues, accumulations of HSP70 chaperones and co-chaperones are also prominent [25,47], and abnormal mitochondrial morphology with the cristae disconnecting from the inner membranes was shown previously by means of electron microscopy [12].

### 4.5. Absence of CLPP Affects the Respiratory Chain

A fifth novel finding of this study in the *P. anserina* CLPP-null proteome profile is the modest accumulation of coherent respiratory complex IV components, together with a minor accumulation of dispersed complex I factors.

The affected complex IV factors were as follows:

B2B1C3 is homologous to the NADH–ubiquinone reductase complex 1 MLRQ subunit (NDUFA4 = COXFA4), an assembly factor and stress adaptor within the cytochrome c oxidase (CIV) complex [127,128,129,130,131,132,133].

B2B7U7 is homologous to the cytochrome c oxidase subunit VII (COX7A), another assembly factor that closely cooperates with NDUFA4, which permits the integration of mt-CO3 and completion of complex IV [134,135,136]. Upon formation of respiratory supercomplexes, COX7A is crucial for the interaction between complex IV and complex I [137].

P20682 is the mitochondrially encoded cytochrome c oxidase subunit 2 (COX2), which contains the dinuclear copper A center; it enters the complex IV assembly process before COX7A and NDUFA4 act [134,138,139]. This might be a specific site of pathology given that excess heme is a known consequence of the absence of CLPP and might cause problems in the assembly of the heme-binding COX1 and the copper-binding COX2, and the downstream completion of complex IV assembly.

The affected complex I components were as follows:

A0A090CEV3 is the NADH–ubiquinone oxidoreductase 9.5 kDa subunit (N9IM, homologous to NDUFA3), which serves in the proximal half of the membrane arm of complex I (P_P_ ND1 module) [140,141]. 

B2AR06 is the NADH dehydrogenase (ubiquinone) alpha subcomplex subunit 1 (NDUFA1), which is part of the proximal half of the membrane arm of complex I (P_P_ ND2 module) [140,141]. 

B2AR53 is homologous to the NADH:ubiquinone oxidoreductase ESSS subunit (NDUFB11), which serves in the distal half of the membrane arm of complex I (P_D_ ND4 module) [140,141]. 

B2B5R1 is the NADH dehydrogenase (ubiquinone) 1 beta subcomplex subunit 2 (NDUFB2, CI-AGGG), which is located in the distal half of the membrane arm of complex I (P_D_ ND5 module) [140,141]. 

B2AWS7 is the NADH dehydrogenase (ubiquinone) 1 alpha subcomplex 6 subunit (homologous to NDUFA6, LYRM6), which is placed in the Q-module before the N-module is added in the assembly of complex I [142,143]. 

B2AT34 is the NADH dehydrogenase (ubiquinone) 1 alpha subcomplex 2 subunit (NDUFA2, CI-B8), which is part of the N-module of complex I [142,143].

B2AYP8 contains a domain of the complex I subunit NDUFS6, which is in the matrix arm of complex I (N-module) [140,141].

Thus, the minor but significant accumulations of complex I subunits are non-specifically distributed across all its membrane and matrix modules. Thus, these fragmentary findings probably reflect a compensatory upregulation of the complete complex I in the face of mitochondrial dysfunction. An accumulation of complex I subunits was not observed in CLPP-null mouse tissues [25]. 

## 5. Conclusions

Overall, it is clear from our *P. anserina* and mouse findings, which were discussed above, that the absence of CLPP has consistent effects throughout eukaryotic evolution:

(1) The consequent accumulation of CLPX with its PLP cofactor not only activates δALA production with downstream heme biosynthesis, but, in parallel, it also reduces the levels of other delta-amino acids such as Arg, Cit, and His. The consistent accumulation of ornithine delta-aminotransferase (OAT) and its probable activation by CLPX-PLP likely contribute to this effect. The halved Arg levels probably explain the growth deficit of CLPP-null mammals, so Arg supplementation in the diet might rescue the short stature and low muscle mass of PRLTS3 patients.
(2) The absence of CLPP with excess CLPX alters the proteins in the LSU central protuberance and L7/L12 stalk, which are key for the processing and integration of tRNA^Val/Phe^ as well as the SSU interactions required for complete mitoribosomal assembly.
(3) Within the mitochondrial protein aggregation pathway, HSP70 accumulation is prominent.
(4) An assembly problem involving the respiratory chain complex IV heme/copper-binding subunits could explain the mouse and *P. anserina* observations.

In summary, despite differences in phenotypic consequences, there is a clear consistency between CLPP-null *P. anserina* and mice regarding dysregulated metabolites and proteome pathways. The elevated abundances of CLPX, ALAS, and OAT are among the most consistent findings in proteome profiles in all the species that have been studied [7]; thus, this metabolic distortion is probably a general feature of CLPP loss.

## Data Availability

All primary data are publicly available via the PeptideAtlas repository with the dataset identifier PASS05856 and the ProteomeXchange Consortium with the dataset identifier PXD048640.

## Supplementary Materials

The following supporting information can be downloaded at: https://www.mdpi.com/article/10.3390/biom14020241/s1, Figure S1: Quantitative immunoblots of WT and CLPP-null cerebella under study verified the absence of CLPP and the >12-fold excess abundance of CLPX in mutant tissues. Table S1: *P. anserina* CLPP-null metabolome profile at day 5 of culture. Table S2: *P. anserina* CLPP-null proteome profile at day 8 of culture. Table S3: *M. musculus* CLPP-null cerebellar metabolome profile, first tier. Table S4: *M. musculus* CLPP-null cerebellar metabolome profile, second tier.

## Abbreviations

2PG	2-phosphoglycerate
5S rRNA	the smallest rRNA within the eukaryotic ribosomal LSU
60S subunit	eukaryotic ribosomal LSU with sedimentation at 60 Svedberg units
AAA+	ATPases associated with various cellular activities
ABAT	4-aminobutyrate aminotransferase
ABC	ammonium bicarbonate
ACTB	beta-actin protein
AGAT	also known as GATM, L-arginine/glycine amidinotransferase
AGC	automatic gain control
AGXT2	alanine–glyoxylate aminotransferase 2
ALA	also known as deltaALA and delta-aminolevulinic acid
Ala	alanine
ALAS	delta-aminolevulinic acid synthase, generic
ALAS1	delta-aminolevulinic acid synthase 1, non-specific
ALAS2	delta-aminolevulinic acid synthase 1, erythroid-specific
ALDH18A1	aldehyde dehydrogenase 18 family member A1
Arg	arginine
ASMT	acetylserotonin O-methyltransferase
Asn	asparagine
Asp	aspartate
ATF4	activating transcription factor 4
ATP	adenosine trisphosphate
BAG-domain	Bcl-2-associated athanogene domain
BCAT2	branched-chain amino acid transaminase 2
BCL2	BCL2 apoptosis regulator
C1/4/5/6	chain composed of 1/4/5/6 carbons
C57BL/6	inbred substrain 6 generated by C.C. Little, from Abbie Lathrop’s mouse 57, with nearly black coat
CI	respiratory chain complex I
CIV	respiratory chain complex IV
Cit	citrulline
CLPA-E	caseinolytic mitoch. matrix peptidase chaperone subunit A-E
CLPP	caseinolytic mitochondrial matrix peptidase proteolytic subunit
CLPX	caseinolytic mitochondrial matrix peptidase chaperone subunit X
CM liquid medium	complete medium containing glucose monohydrate
Co^2+^	elemental cobalt as divalent cation
CoA	coenzyme A
COX2	mitochondrially encoded cytochrome C oxidase II
COX7A	cytochrome C oxidase subunit 7A1
COXFA4	cytochrome C oxidase subunit FA4, also known as NDUFA4
CPOX	coproporphyrinogen oxidase
CPT	carnitine palmitoyltransferase 2
CTP	cytidine trisphosphate
Cys	cysteine
DNA	desoxyribonucleic acid
DnaK	*E. coli* chaperone protein
DTT	dithiothreitol
EARS2	glutamyl-tRNA synthetase 2, mitochondrial
eIF6	eukaryotic translation initiation factor 6
ETNPPL	ethanolamine-phosphate phospholyase
ESI	electrospray ionization
ESSS	=NDUFB11, NADH-ubiquinone oxidoreductase subunit B11
FAD	flavin adenine dinucleotide
FC	fold change
FDR	false discovery rate
Fe^2+^	ferrous iron = iron(II), elemental iron as divalent cation
FECH	ferrochelatase
FeS clusters	iron–sulfur clusters
G1SA	glutamate-1-semialdehyde, also known as GSA
G5SA	glutamate-5-semialdehyde
GABA	gamma-amino-butyric acid
GAMT	guanidinoacetate N-methyltransferase
GAR1	Gar1 ribonucleoprotein homolog
GATC	glutaminyl-tRNA synthase subunit C, mitochondrial
GATM	glycine amidinotransferase
GDH1	glutamate decarboxylase 1
GFM1/2	translation elongation factor G, mitochondrial, variant 1/2
GH	growth hormone
GLDC	glycine decarboxylase
Gln	glutamine
GltX	glutamate-tRNA ligase
Glu	glutamate
Gly	glycine
GO-term	gene ontology term
GOT1/2	glutamic-oxaloacetic transaminase 1/2
GPT	glutamic-pyruvic transaminase
GSSG	glutathione disulfide
GTP	guanosine triphosphate
H/ACA	sequence motifs H box (consensus ANANNA) and ACA box (ACA)
HARS2	histidine-tRNA ligase, mitochondrial
HemA	glutamyl-tRNA reductase
HemL	glutamate-1-semialdehyde 2,1-aminomutase
Hexose-1,6BP	hexose-1,6-bisphosphate
Hexose-6P	hexose-6-phosphate
His	histidine
Hsc70	heat shock cognate 71 kDa protein
Hse	homoserine
HSP70	heat shock protein family A (Hsp70) member 4
i-AAA	mitochondrial intermembrane space AAA+ protease
Ile	isoleucine
kDa	kiloDalton (molecular weight unit)
L7/L12 stalk	stalk structure in the mitoribosomal LSU, with proteins 7/12
LARS2	leucyl-tRNA synthetase 2, mitochondrial
LC/MS	liquid chromatography/mass spectrometry
Leu	leucine
LMBRD1	lysosomal cobalamin transport escort protein LMBR1
LonP	Lon peptidase 1 homolog, mitochondrial
Lsm7	like-SM domain-containing protein 7, cytosolic
LSU	mitoribosomal large subunit
LYRM6	protein 6 with conserved tripeptide (LYR) motif
m3G	2,2,7-trimethyl guanosine
MCX1	yeast CLPX homolog
Met	methionine
Mg^2+^	elemental magnesium as divalent cation
1-MHis	1-methylhistamine
MRM	multiple reaction mode
MRPP1	mitochondrial ribonuclease P protein 1, also known as TRMT10C
MRPP3	mitochondrial ribonuclease P protein 3, also known as PRORP
MS	mass spectrometry
MRPL30	large ribosomal subunit protein uL30m
MRPL33	large ribosomal subunit protein bL33m
MTBE	methyl-tert-butyl ester
mt-CO3	mitochondrially encoded cytochrome C oxidase III
mtDNA	mitochondrial DNA, nucleoid
5,10-MTHF	5,10-methylenetetrahydrofolate
MTHFD2	methylenetetrahydrofolate dehydrogenase (NADP+ dependent) 2, methenyltetrahydrofolate cyclohydrolase
NADPH	nicotinamide adenine dinucleotide phosphate
NDUFA1	NADH:ubiquinone oxidoreductase subunit A1
NDUFA2	NADH:ubiquinone oxidoreductase subunit A2
NDUFA2	NADH:ubiquinone oxidoreductase subunit A3
NDUFA4	NADH:ubiquinone oxidoreductase subunit A4
NDUFA6	NADH:ubiquinone oxidoreductase subunit A6
NDUFB2	NADH:ubiquinone oxidoreductase subunit B2
NDUFB11	NADH:ubiquinone oxidoreductase subunit B11
NDUFS6	NADH:ubiquinone oxidoreductase subunit S6
NFS1	nitrogen fixing bacteria S-like protein 1, cysteine desulfurase
NOP10	homolog of yeast Nop10p
NTMT1/2	N-terminal Xaa-Pro-Lys N-methyltransferase 1/2
OAT	ornithine aminotransferase
ODC	also known as ODC1, ornithine decarboxylase 1
ODO1	2-oxoglutarate dehydrogenase complex component E1
ONC201	=dordaviprone: 11-benzyl-7-[(2-methylphenyl)methyl]-2,5,7,11-tetrazatricyclo [7.4.0.02,6]trideca-1(9),5-dien-8-one
Orn	ornithine
OTC	ornithine transcarbamylase
P5C	delta-1-pyrroline-5-carboxylate
P5CDH	pyrroline-5-carboxylate dehydrogenase
P5CS	pyrroline-5-carboxylate synthetase, also known as ALDH18A1
P5P	pyridoxal-5′-phosphate
Pa	*Podospora anserina* fungus
PADI	peptidylarginine deiminases
PAGE	polyacrylamide gel electrophoresis
*PaIap*	=B2B020 in UniProt; mitoch. intermembrane space AAA+ protease
*PaSnf1*	=B2B4C1 in UniProt; sucrose non-fermenting complex, catalytic 1
PEP	phosphoenolpyruvate
1,3PG	1,3-bisphospho-glycerate
2PG	2-phosphoglycerate
3PG	3-phosphoglycerate
PHD	prolyl-3-hydroxlase domain
Phe	phenylalanine
PHYKPL	5-phosphohydroxy-L-lysine phospholyase
PLP	pyridoxal-5′-phosphate
PodAns	*Podospora anserina* fungus
PP/PE	polypropylene/polyethylene
PPIX	protoporphyrinogen IX
PRLTS3	Perrault syndrome type 3
Pro	proline
PRORP	protein-only RNase P catalytic subunit
RMND1	required for meiotic nuclear division 1 homolog
RNA	ribonucleic acid
rRNA	ribosomal RNA
SAM	S-adenosyl methionine
SAM-MTases	S-adenosyl methionine-dependent methyltransferases
SAP domain	DNA-binding 35-residue motif, named after SAF-A/B, acinus, andPIAS, three proteins known to contain it
SDS	sodium dodecyl sulfate
Ser	serine
SHMT2	serine hydroxymethyltransferase 2, mitochondrial
Sm domain	occurs in Sm proteins, named in honor of patient Stephanie Smith
SmG-like domain	spliceosomal core protein SmG, binds to AU dinucleotide
snoRNA	small nucleolar *RNA*
snRNA	small nuclear RNA
snRNP	small nuclear ribonucleoprotein
SSU	ribosomal small subunit
STRING	search tool for the retrieval of interacting genes/proteins
Tae1	alpha N-terminal protein methyltransferase 1
TCA cycle	tricarboxylic acid cycle
Thr	threonine
TRMT10C	tRNA methyltransferase 10C, mitochondrial RNase P subunit
tRNA	transfer RNA
tRNA^Val/Phe^	transfer RNA for valine or phenylalanine
Trp	tryptophan
TWNK	twinkle
Tyr	tyrosine
UniProt	public database about proteins, unifies nomenclature
UniProt-ID	UniProt protein identifier number
UPR^mt^	mitochondrial unfolded protein response
UTP	uridine triphosphate
*v*/*v*	volume per volume
WT	Wild-type
ZFE	Zentrale Forschungs-Einrichtung
