# Supplementary figures and images for: CLPP-Null Eukaryotes with Excess Heme Biosynthesis Show Reduced L-arginine Levels, Probably via CLPX-Mediated OAT Activation
Source: Biomolecules. 2024 Feb 19;14(2):241. doi: 10.3390/biom14020241 (PMC10886707; doi:10.3390/biom14020241)

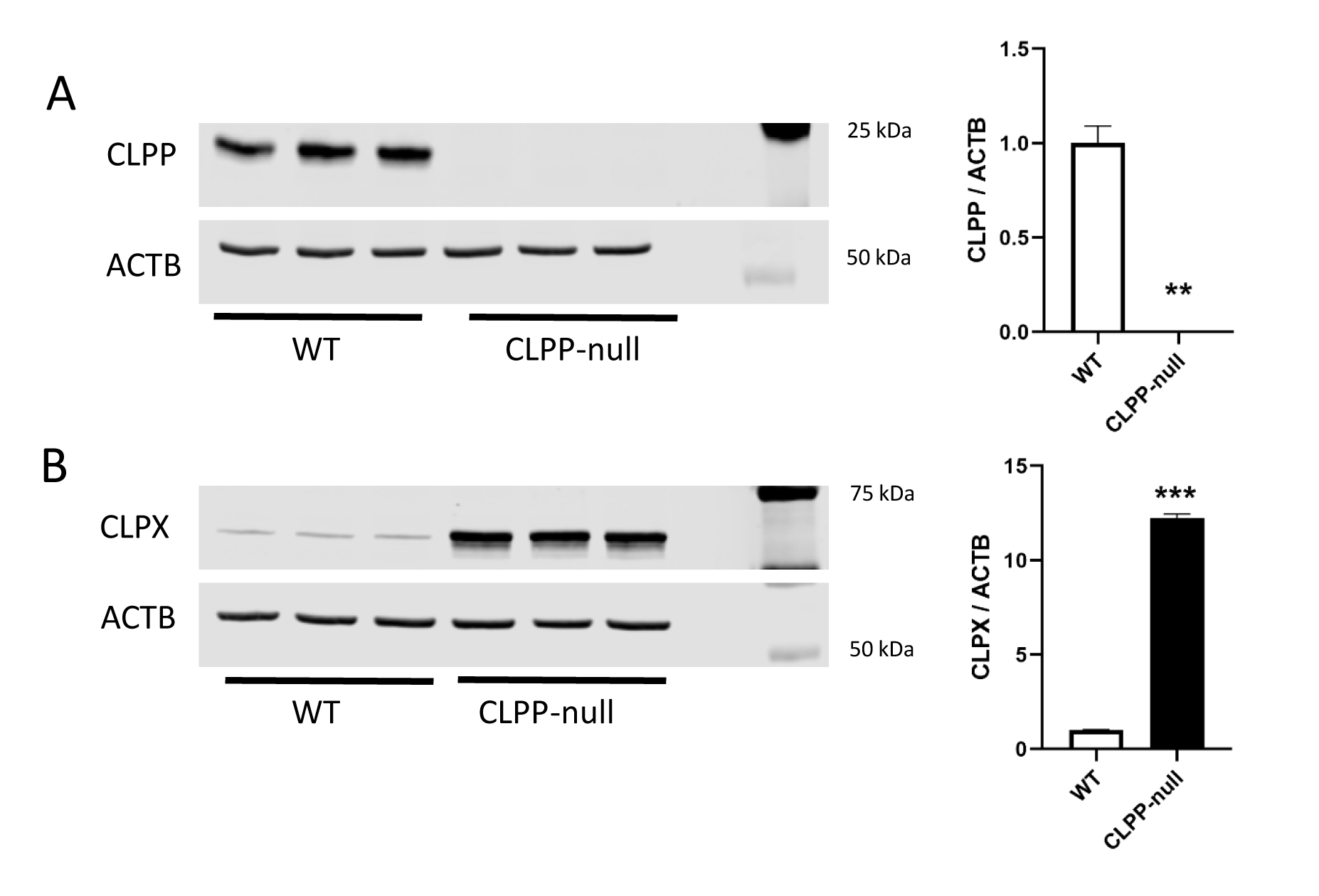

Supplement: Supplementary file 1 [file biomolecules-14-00241-s001.zip › Suppl_Fig_S1.tif]
